# Mediation analysis of the causal pathway from gastroesophageal reflux disease to pancreatitis: A Mendelian randomization study

**DOI:** 10.1097/MD.0000000000049599

**Published:** 2026-07-17

**Authors:** Jinfu Zeng, Zhenhua Huang, Jinqiang Zhang

**Affiliations:** a Department of Thoracic and Cardiovascular Surgery, Pingxiang People’s Hospital, Pingxiang, China.

**Keywords:** cholelithiasis, gastroesophageal reflux disease, mediation analysis, Mendelian randomization, pancreatitis

## Abstract

Although gastroesophageal reflux disease (GERD) and pancreatitis are both common gastrointestinal disorders, few studies have investigated their potential causal relationship. Clarifying whether GERD contributes to the development of acute pancreatitis (AP) and chronic pancreatitis (CP), and the possible mediating role of cholelithiasis, may provide new insights into shared pathogenic mechanisms. A univariable Mendelian randomization (UVMR) analysis was performed to assess the causal effects of GERD on AP and CP. To improve statistical power, the results from the discovery and replication cohorts were combined through meta-analysis. Multivariable Mendelian randomization (MVMR) was subsequently applied to adjust for the potential confounding effect of cholelithiasis. Finally, mediation analysis was conducted to evaluate the mediating role of cholelithiasis in the causal pathway from GERD to AP and CP. Multiple analyses, including heterogeneity analysis and horizontal pleiotropy analysis, were performed for sensitivity assessment. This study provides genetic evidence supporting a causal relationship between GERD and pancreatitis. The findings further highlight cholelithiasis as a partial mediator in this pathway, offering novel insights into shared mechanisms and potential preventive strategies for pancreatitis. UVMR suggested a positive causal association between GERD and both AP (discovery cohort: odds ratio [OR], 1.46; 95% confidence interval [CI], 1.29–1.65; *P* < .001; replication cohort: OR, 1.63; 95% CI, 1.37–1.93; *P* < .001) and CP (discovery cohort: OR, 1.47; 95% CI, 1.24–1.73; *P* < .001; replication cohort: OR, 1.50; 95% CI, 1.15–1.96; *P* = .003). Meta-analysis confirmed consistent and robust causal estimates across the discovery and replication datasets (AP: pooled OR, 1.52; 95% CI, 1.37–1.68; *P* < .001; CP: pooled OR, 1.48; 95% CI, 1.28–1.70; *P* < .001). After adjustment for cholelithiasis using MVMR, the causal effects were attenuated but remained statistically significant (AP: OR, 1.24; 95% CI, 1.06–1.44; *P* = .006; CP: OR, 1.39; 95% CI, 1.12–1.72; *P* = .003). Mediation analysis indicated that cholelithiasis partially mediated the causal effect of GERD on AP (mediation proportion [95% CI]: 42.4% [27.7–57.2%], *P* < .001) and CP (mediation proportion [95% CI]: 21.9% [0.1–43.7%], *P* = .048), suggesting that gallstone formation may be an important biological link between reflux disease and pancreatic inflammation. No significant evidence was detected in the sensitivity analyses (All *P* > .050).

## 1. Introduction

Gastroesophageal reflux disease (GERD) is one of the most common upper gastrointestinal disorders worldwide,^[1,2]^ characterized by the reflux of gastric contents into the esophagus, leading to symptoms such as heartburn and regurgitation.^[3-5]^ In addition to local esophageal injury, GERD has been increasingly recognized as a systemic condition associated with various extra-esophageal diseases,^[6,7]^ including respiratory,^[8,9]^ cardiovascular,^[10,11]^ and metabolic disorders.^[12,13]^ Recent clinical observations have suggested a potential link between GERD and pancreatitis^[14]^; however, the nature of this relationship remains poorly understood.

Pancreatitis, including acute pancreatitis (AP) and chronic pancreatitis (CP),^[15,16]^ represents a major cause of gastrointestinal morbidity and mortality globally. The disease involves pancreatic inflammation and progressive structural damage,^[17]^ leading to long-term complications such as exocrine insufficiency and diabetes.^[18]^ While cholelithiasis, alcohol consumption, and metabolic disturbances are well-established risk factors for pancreatitis,^[19-21]^ accumulating evidence suggests that other gastrointestinal conditions may also contribute to its onset and progression. GERD and pancreatitis share several risk determinants, including obesity, high-fat diet, metabolic disturbances, diabetes, and systemic inflammatory activation, implying possible overlapping pathophysiological pathways.

Despite these clinical associations, few studies have directly examined whether GERD exerts a causal effect on the risk of pancreatitis. Observational studies are often confounded by shared lifestyle and metabolic factors, making it difficult to disentangle causality from correlation. Mendelian randomization (MR) offers a powerful approach to overcome these limitations by using genetic variants as instrumental variables (IVs) to infer causality between an exposure and an outcome.^[22,23]^ This method minimizes bias from confounding and reverse causation, providing more reliable causal estimates.

In addition, cholelithiasis – a major etiological factor for pancreatitis – may act as an intermediary in this pathway. GERD and gallstone disease share common metabolic and dietary risk factors, and bile reflux could theoretically influence pancreatic inflammation. However, whether cholelithiasis mediates the effect of GERD on pancreatitis has not been clarified. Therefore, this study aimed to investigate the causal relationship between GERD and pancreatitis using a 2-sample MR framework. Univariable Mendelian randomization (UVMR) was first performed to estimate the direct causal effects of GERD on AP and CP, followed by multivariable Mendelian randomization (MVMR),^[24,25]^ which was applied to adjust for the potential confounding influence of cholelithiasis. Finally, mediation analysis was conducted to evaluate the mediating role of cholelithiasis in the causal pathway from GERD to pancreatitis. This integrated genetic approach provides new evidence on the complex interaction between upper gastrointestinal disorders and pancreatic inflammation.

## 2. Materials and methods

### 2.1. Study design

**Figure 1.**
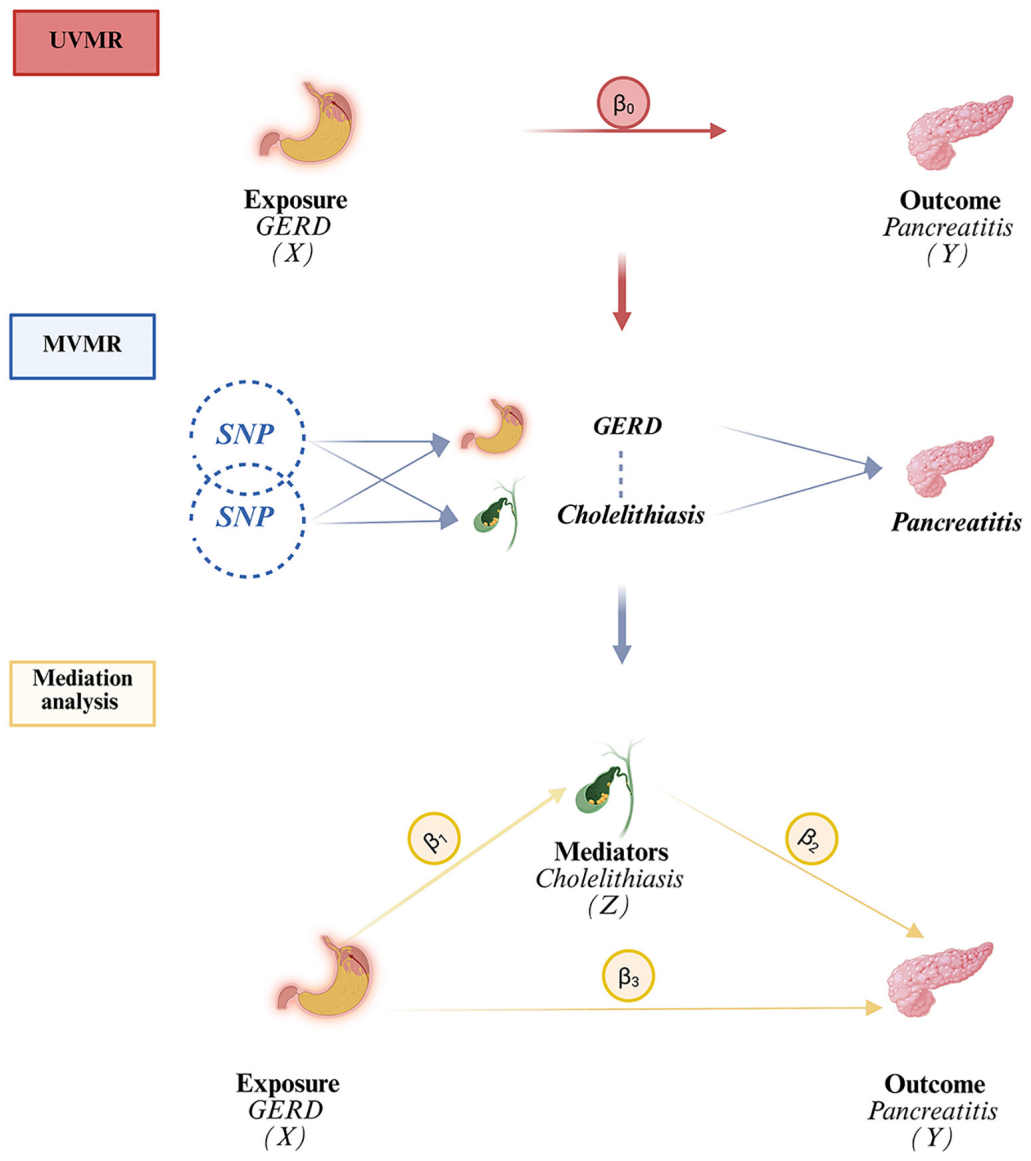
Schematic diagram of this MR study exploring the causal effect of GERD on pancreatitis. GERD = gastroesophageal reflux disease, MR = Mendelian randomization, MVMR = multivariable Mendelian randomization, SNP = single nucleotide polymorphism, UVMR = univariable Mendelian randomization.

Based on the core assumptions of the MR study,^[26]^ UVMR was first conducted to evaluate the causal effect of GERD on AP and CP. Subsequently, MVMR was applied to estimate the independent effect of GERD on pancreatitis after accounting for the potential confounding influence of cholelithiasis. Finally, mediation analysis was performed to investigate the extent to which cholelithiasis mediates the causal pathway from GERD to pancreatitis (Fig. 1).

### 2.2. Data sources

The pancreatitis genome-wide association study (GWAS) data for the discovery cohort (AP: ID, finngen_R12_K11_ACUTPANC; sample size = 602,604; CP: ID, finngen_R12_K11_CHRONPANC; sample size = 442,238) were obtained from the latest release of the FinnGen database (https://www.finngen.fi/en),^[27]^ while the GWAS summary statistics for GERD (ID, ebi-a-GCST90000514; sample size = 602,604), cholelithiasis(ID, ebi-a-GCST90013889; sample size = 404,405), and the replication pancreatitis cohort (AP: ID, ebi-a-GCST90018789; sample size = 479,902; CP: ID, ebi-a-GCST90018821; sample size = 477,528)were obtained from the Integrative Epidemiology Unit (https://gwas.mrcieu.ac.uk/). All GWAS populations were of European ancestry. Detailed information on the GWAS datasets used in this MR study is summarized in Table 1.

**Table 1:** Details of the summary GWAS datasets used for this MR analysis.

Trait	ID of GWAS	Sample size	Consortium (yr)	Population
GERD	ebi-a-GCST90000514	602604	IEU (2021)	European
AP (discovery cohort)	finngen_R12_K11_ACUTPANC	445864	FinGen (2024)	European
AP (replication cohort)	ebi-a-GCST90018789	479902	IEU (2021)	European
CP (discovery cohort)	finngen_R12_K11_CHRONPANC	442238	FinGen (2024)	European
CP (replication cohort)	ebi-a-GCST90018821	477528	IEU (2021)	European
Cholelithiasis	ebi-a-GCST90013889	404405	IEU (2021)	European

AP = acute pancreatitis, CP = chronic pancreatitis, GERD = gastroesophageal reflux disease, GWAS = genome-wide association study, IEU = Integrative Epidemiology Unit.

### 2.3. Selection of IVs

**Figure 2.**
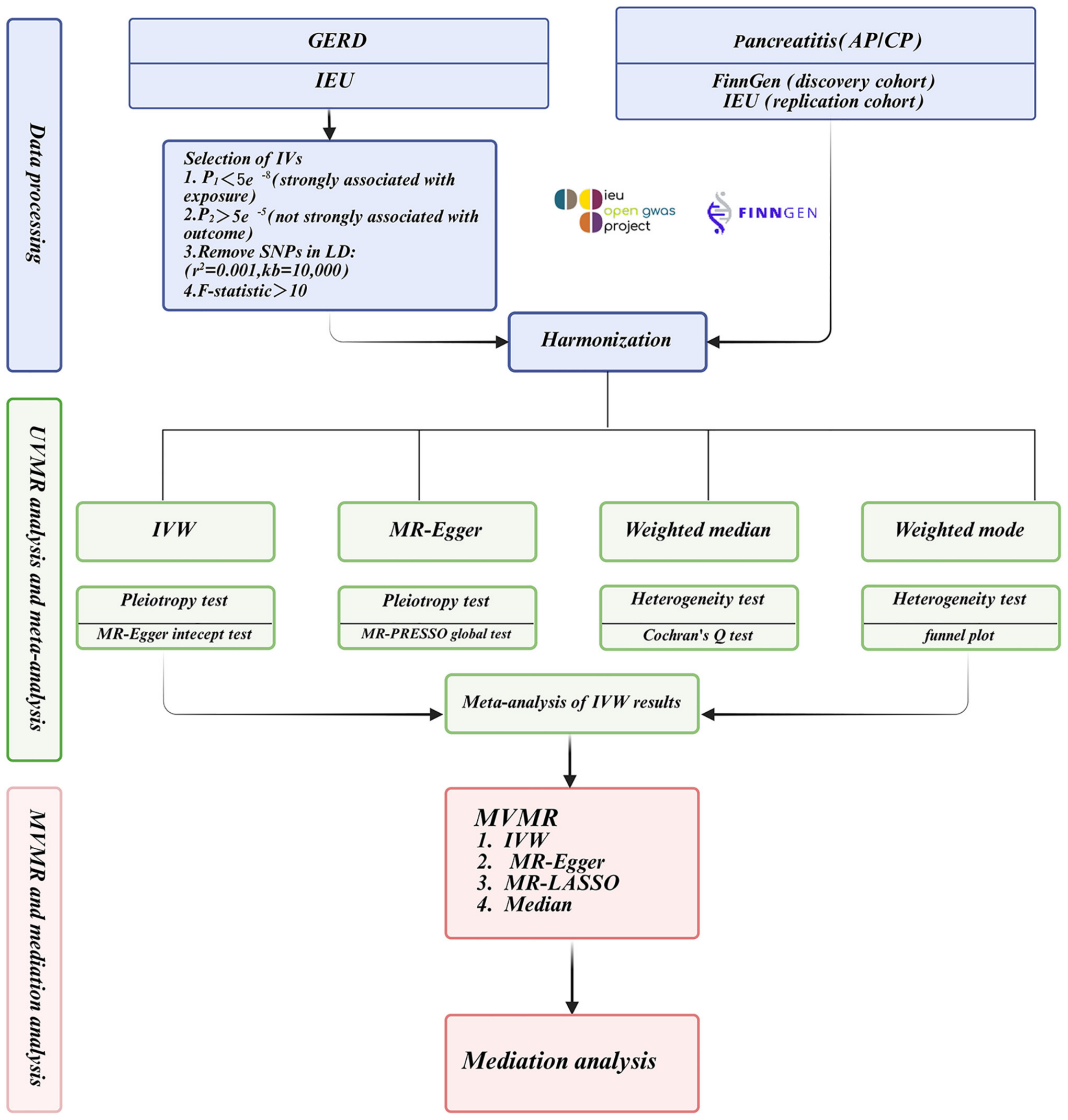
Flowchart of this MR study exploring the causal effect of GERD on pancreatitis. AP = acute pancreatitis, CP = chronic pancreatitis, GERD = gastroesophageal reflux disease, IEU = integrative epidemiology unit, IV = instrumental variables, IVW = inverse-variance weighted, LASSO = least absolute shrinkage and selection operator, LD = linkage disequilibrium MR-Egger = Mendelian randomization-Egger, MVMR = multivariable Mendelian randomization, MR-PRESSO = Mendelian randomization pleiotropy residual sum and outlier, SNP = single nucleotide polymorphism, UVMR = univariable Mendelian randomization.

**Figure 3.**
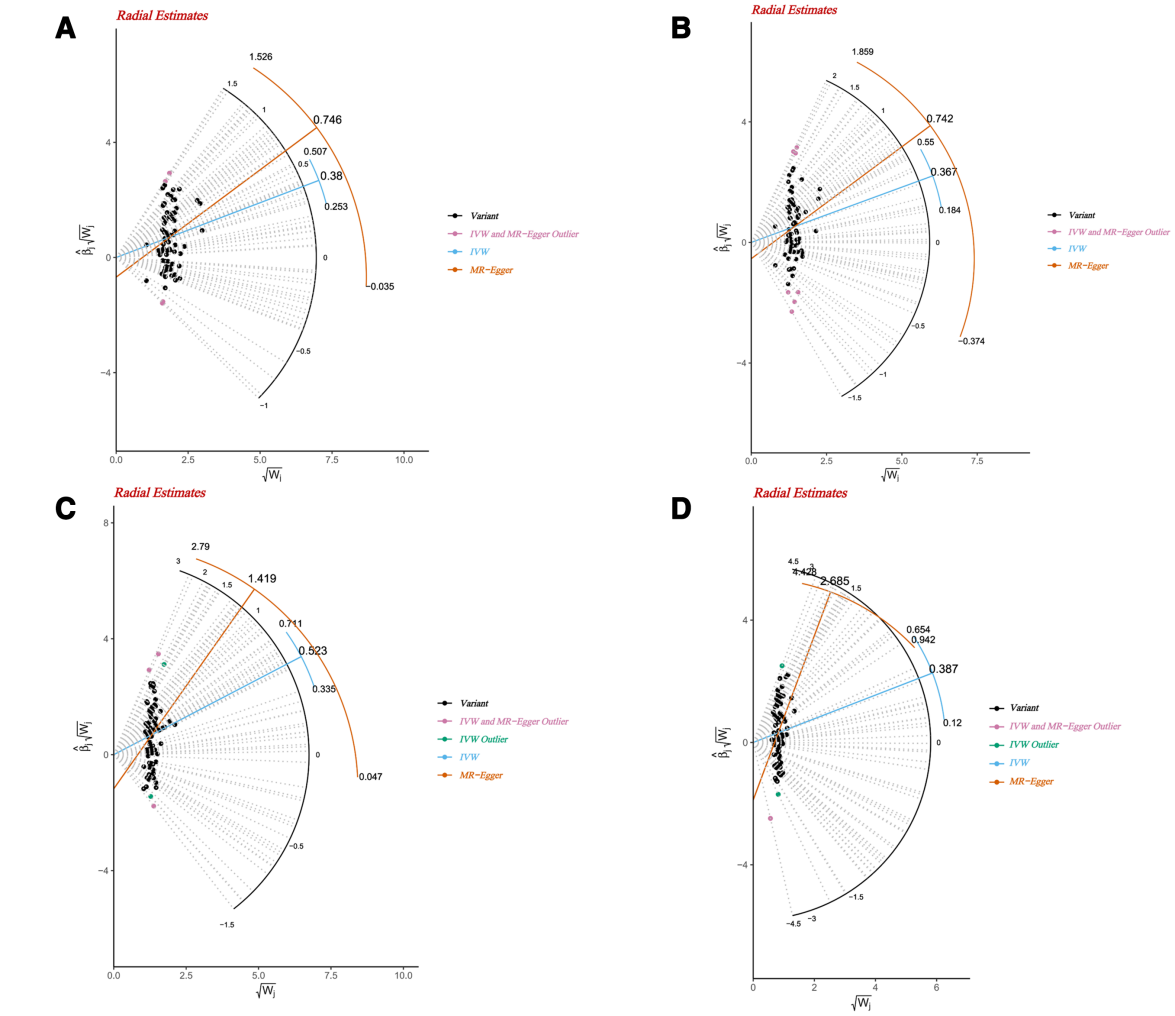
Radial plots to visually depict individual outlier SNPs by RadialMR in this MR analysis exploring the causal effect of GERD on pancreatitis. (A) Radial plot to visually depict individual outlier SNPs by RadialMR in this MR analysis exploring the causal effect of GERD on AP in the discovery cohort. (B) Radial plot to visually depict individual outlier SNPs by RadialMR in this MR analysis exploring the causal effect of GERD on AP in the replication cohort. (C) Radial plot to visually depict individual outlier SNPs by RadialMR in this MR analysis exploring the causal effect of GERD on CP in the discovery cohort. (D) Radial plot to visually depict individual outlier SNPs by RadialMR in this MR analysis exploring the causal effect of GERD on CP in the replication cohort. Valid SNPs are indicated by black dots, while outlier SNPs are represented by colored dots. AP = acute pancreatitis, CP = chronic pancreatitis, GERD = gastroesophageal reflux disease, IVW = inverse-variance weighted method, MR = Mendelian randomization, SNP = single nucleotide polymorphism.

Single nucleotide polymorphisms (SNPs) strongly associated with GERD were initially selected using a genome-wide significance threshold of *P* < 5 × 10^−8^ (Fig. 2). To minimize the influence of linkage disequilibrium, SNPs were clumped with an *r*^2^ threshold of 0.001 and a window of 10,000 kb. SNPs with an *F*-statistic ≤ 10 were excluded to remove weak instruments [*F* ≈ (β/SE)^2^].^[28]^ Outlier SNPs were subsequently identified using RadialMR^[29]^ (Fig. 3, Table 2), and reverse-direction SNPs were filtered out based on the MR Steiger test.^[30]^ The remaining SNPs were retained as genetic instruments for GERD and used in subsequent MR analyses. Detailed information on the IVs is summarized in Table S1, Supplemental Digital Content 1.

**Table 2:** Outlier SNPs identified by RadialMR.

Outcome (GWAS ID)	Outlier SNPs
AP (finngen_R12_K11_ACUTPANC)	rs12357321; rs4713692; rs569356; rs903678
AP (ebi-a-GCST90018789)	rs11953061; rs1334297; rs9373363; rs1510719; rs903959
CP (finngen_R12_K11_CHRONPANC)	rs12598916; rs1937450; rs2744961; rs6711584; rs7241572; rs9396740; rs942065
CP (ebi-a-GCST90018821)	rs2106353; rs3766823; rs7541875

AP = acute pancreatitis, CP = chronic pancreatitis, GERD = gastroesophageal reflux disease, GWAS = genome-wide association study, SNP = single nucleotide polymorphism.

### 2.4. Statistical analysis

In the UVMR analysis, the inverse-variance weighted (IVW)^[31]^ method served as the primary approach to estimate the causal effect of GERD on pancreatitis, supplemented by MR-Egger,^[32]^ weighted median,^[33]^ and weighted mode^[34]^ methods to ensure result robustness. In the MVMR analysis, the causal association between GERD and AP was further evaluated after adjusting for potential confounding effects of cholelithiasis using IVW, MR-Egger, least absolute shrinkage and selection operator,^[35]^ and median methods. To assess heterogeneity among IVs, Cochran’s *Q* test was performed. Horizontal pleiotropy was evaluated using both the MR-PRESSO global test and the MR-Egger intercept test. Additionally, funnel plots were generated to visually inspect the symmetry of SNP distributions, providing an intuitive assessment of potential heterogeneity or directional bias. A 2-sided *P* value < .05 was considered statistically significant.

## 3. Results

### 3.1. The causal effect of GERD on pancreatitis in UVMR

**Figure 4.**
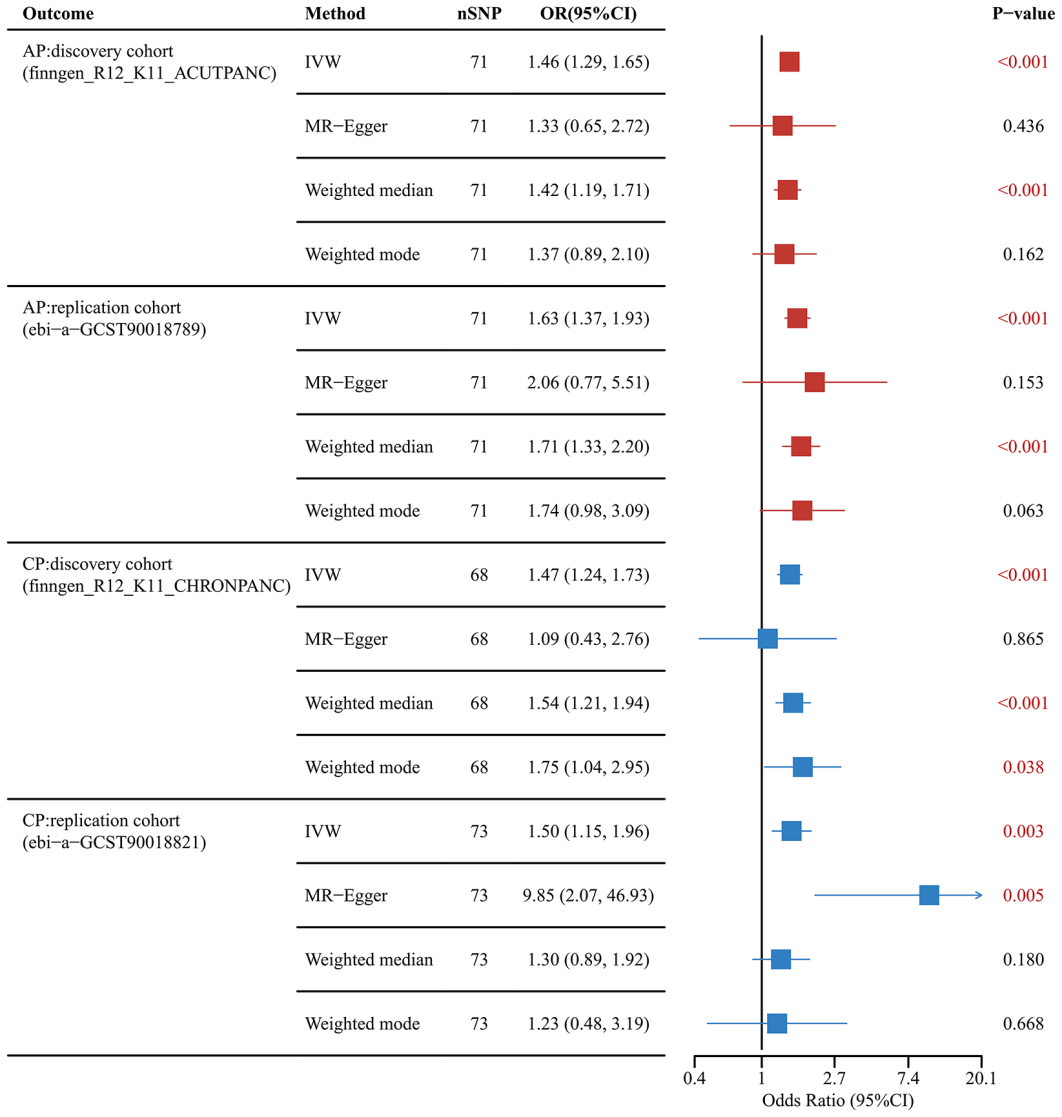
Forest plot illustrating the causal effect of GERD on pancreatitis in UVMR. AP = acute pancreatitis, CI = confidence interval, CP= chronic pancreatitis, GERD = gastroesophageal reflux disease, IVW = inverse-variance weighted method, OR = odds ratio, SNP = single nucleotide polymorphism, UVMR = univariable Mendelian randomization.

**Figure 5.**
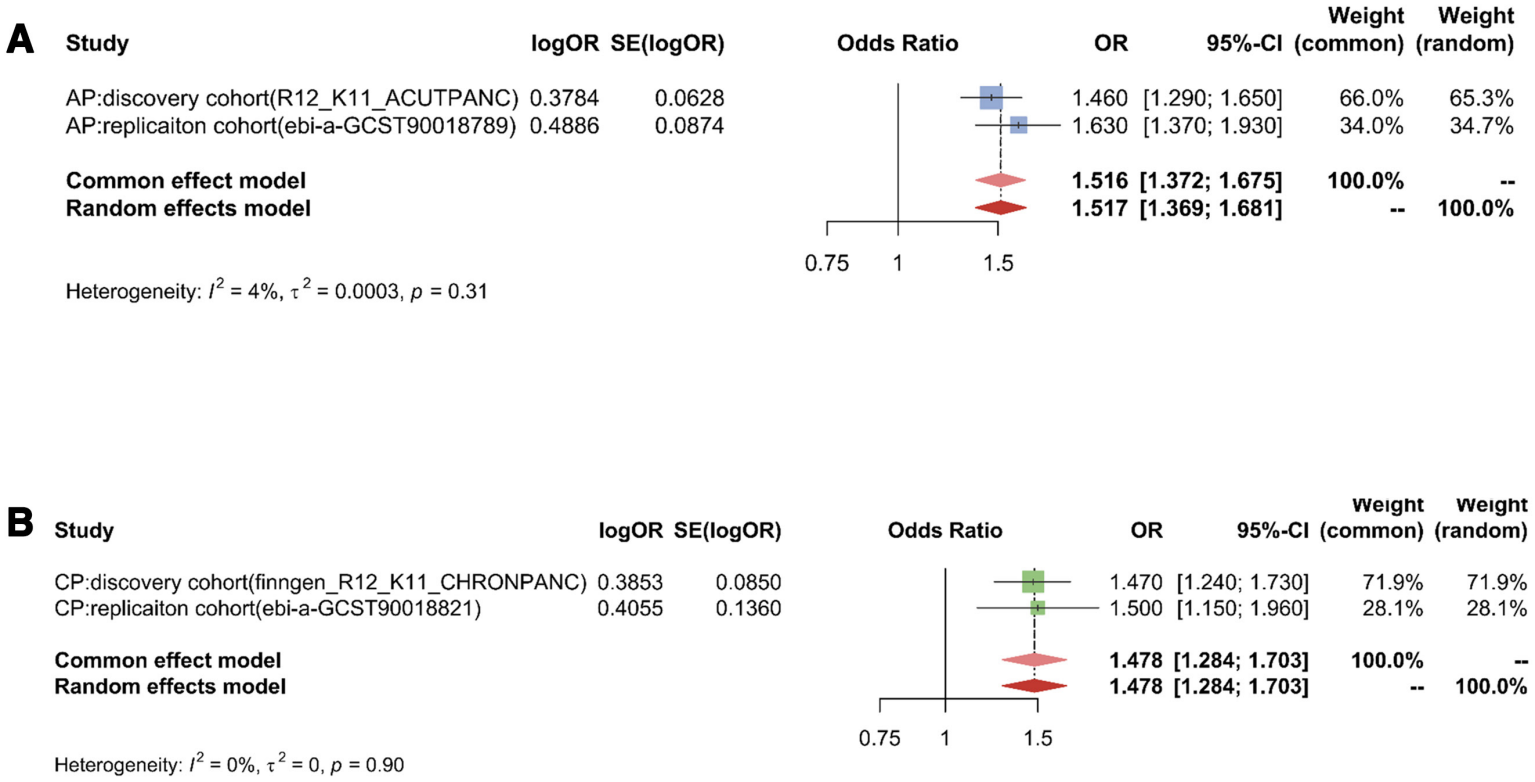
Forest plot illustrating the meta-analysis of causal effects of GERD on pancreatitis in the discovery and replication cohorts in UVMR. (A) Forest plot illustrating the meta-analysis of causal effects of GERD on AP in the discovery and replication cohorts in UVMR. (B) Forest plot illustrating the meta-analysis of causal effects of GERD on CP in the discovery and replication cohorts in UVMR. AP = acute pancreatitis, CI = confidence interval, CP = chronic pancreatitis, GERD = gastroesophageal reflux disease, OR = odds ratio, SE = standard deviation, UVMR = univariable Mendelian randomization.

The UVMR analysis revealed a significant positive causal relationship between GERD and both subtypes of pancreatitis. In the discovery cohort, GERD was found to increase the risk of AP (odds ratio [OR], 1.46; 95% confidence interval [CI], 1.29–1.65; *P* < .001) and CP (OR, 1.47; 95% CI, 1.24–1.73; *P* < .001). These findings were consistently replicated in the replication cohort (AP: OR, 1.63; 95% CI, 1.37–1.93; *P* < .001; CP: OR, 1.50; 95% CI, 1.15–1.96; *P* = .003; Fig. 4), indicating strong reproducibility across independent datasets. The pooled estimates from meta-analysis further strengthened these results, showing robust and consistent associations between GERD and pancreatitis in both datasets (AP: pooled OR 1.52; 95% CI, 1.37–1.68; *P* < .001; CP: pooled OR 1.48; 95% CI, 1.28–1.70; *P* < .001; Fig. 5). These findings suggest that GERD may serve as a potential causal risk factor for the development of both AP and CP.

### 3.2. The causal effect of GERD on pancreatitis in MVMR

**Figure 6.**
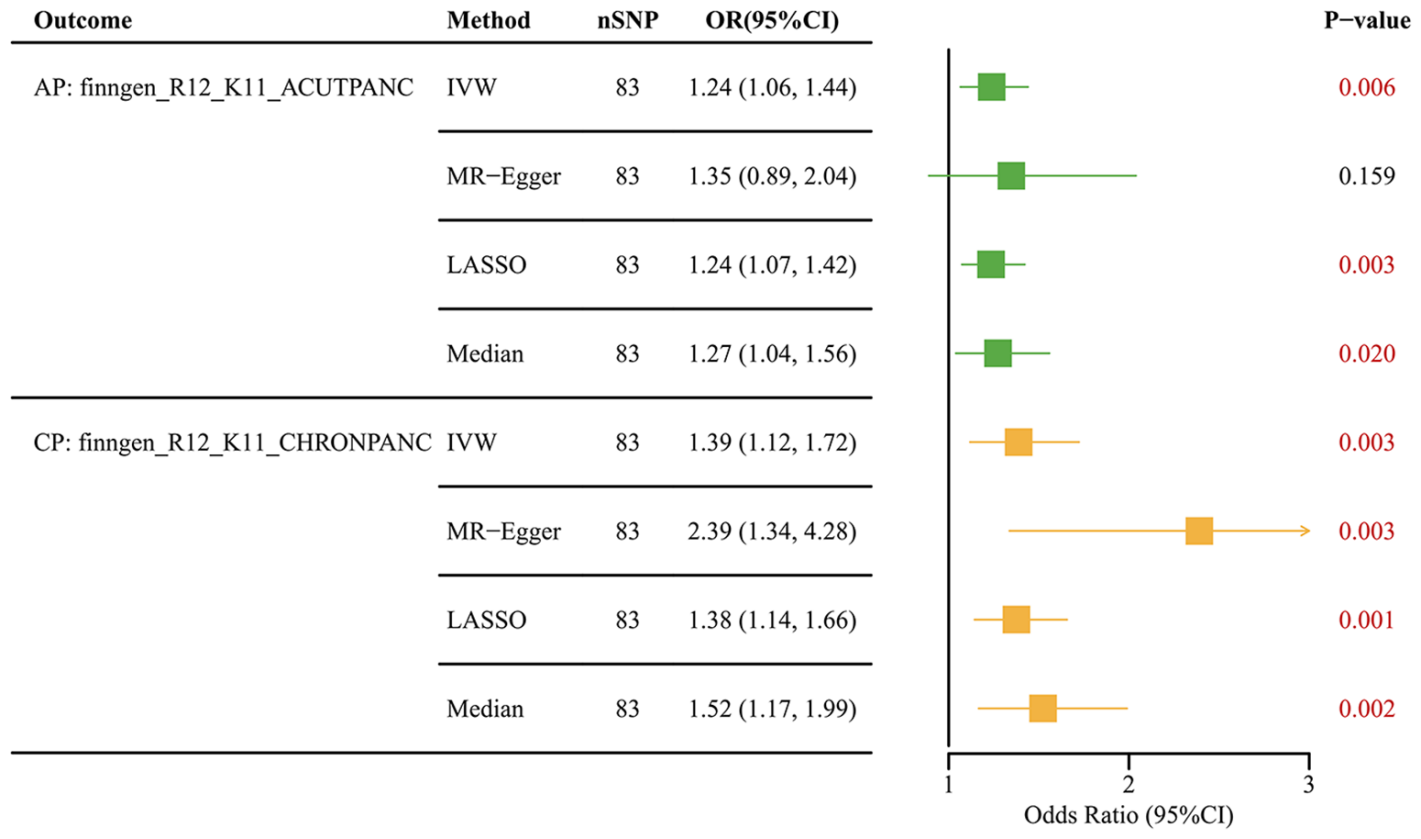
Forest plot illustrating the causal effect of GERD on pancreatitis in MVMR, adjusting for the effect of cholelithiasis. AP = acute pancreatitis, CI = confidence interval, CP = chronic pancreatitis, GERD = gastroesophageal reflux disease, MVMR = multivariable Mendelian randomization, OR = odds ratio.

After adjusting for the potential confounding effect of cholelithiasis using MVMR, the causal effects of GERD on both AP and CP were somewhat attenuated but remained statistically significant. Specifically, for AP, the adjusted OR was 1.24 (95% CI, 1.06–1.44; *P* = .006), and for CP, the adjusted OR was 1.39 (95% CI, 1.12–1.72; *P* = .003; Fig. 6). These findings indicate that although part of the effect of GERD on pancreatitis may be mediated through gallstone disease, GERD independently contributes to the risk of both AP and CP.

### 3.3. Mediation analysis of cholelithiasis in the causal pathway from GERD to pancreatitis

**Figure 7.**
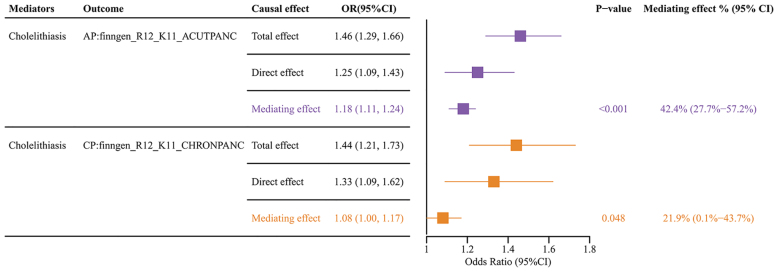
Forest plot illustrating the mediating effect of cholelithiasis on the causal relationship between GERD and pancreatitis. AP = acute pancreatitis, CI = confidence interval, CP, chronic pancreatitis, GERD, gastroesophageal reflux disease, OR = odds ratio.

Mediation analysis revealed that cholelithiasis partially mediated the causal effect of GERD on both AP and CP. For AP, the mediation proportion was 42.4% (95% CI, 27.7–57.2%; *P* < .001), indicating that nearly half of GERD’s effect on acute pancreatitis may be explained through gallstone formation. For CP, the mediation proportion was 21.9% (95% CI, 0.1–43.7%; *P* = .048; Fig. 7), suggesting a smaller but significant indirect effect through cholelithiasis. These findings indicate that cholelithiasis contributes to the GERD-pancreatitis pathway.

### 3.4. Sensitivity analysis in this MR analysis

**Figure 8.**
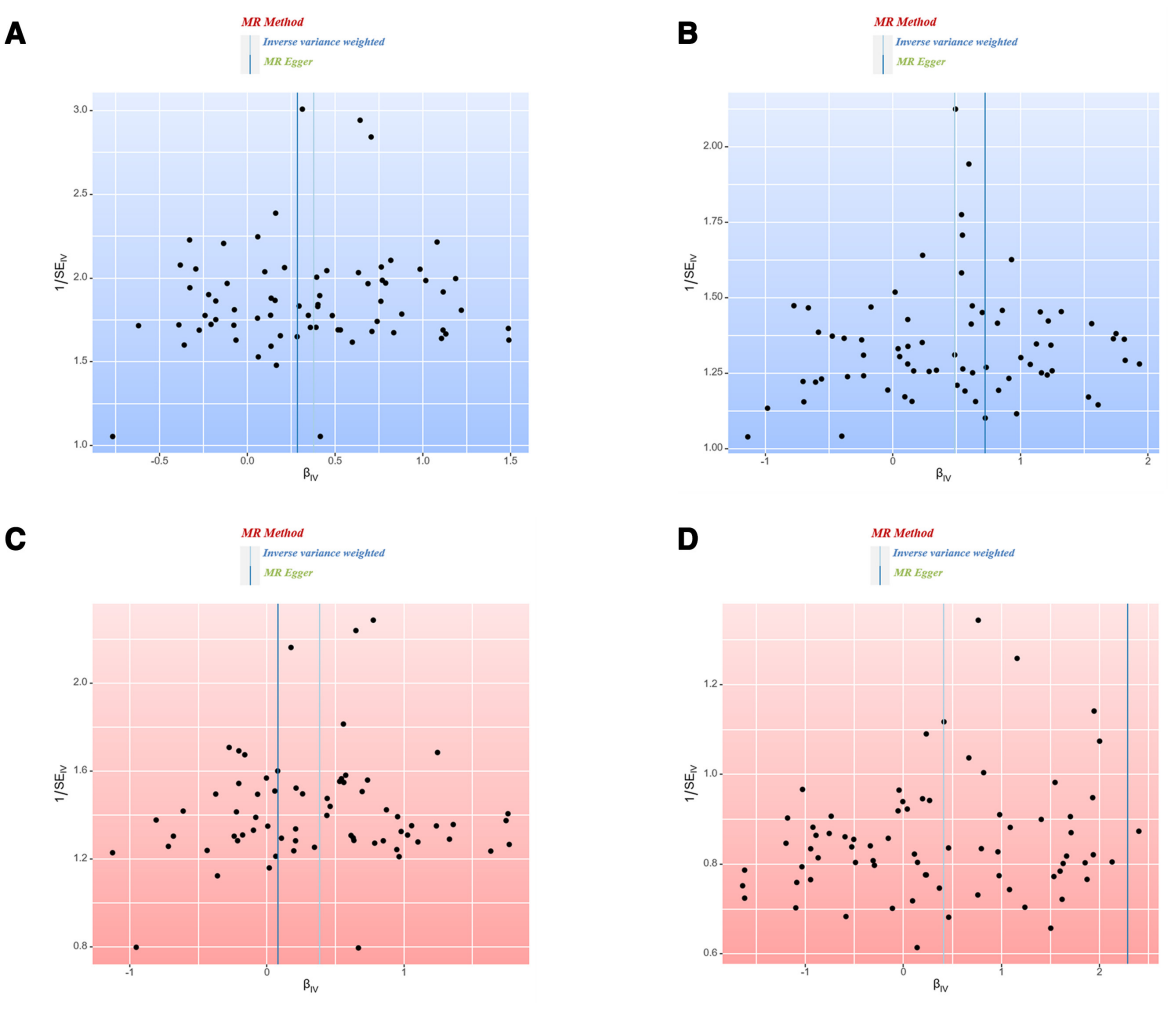
Funnel plots illustrating the heterogeneity of SNPs in this MR study. (A) Funnel plot illustrating the heterogeneity of SNPs in this MR study when exploring the causal effect of GERD on AP in the discovery cohort. (B) Funnel plot illustrating the heterogeneity of SNPs in this MR study when exploring the causal effect of GERD on AP in the replication cohort. (C) Funnel plot illustrating the heterogeneity of SNPs in this MR study when exploring the causal effect of GERD on CP in the discovery cohort. (D) Funnel plot illustrating the heterogeneity of SNPs in this MR study when exploring the causal effect of GERD on CP in the replication cohort gastroesophageal reflux disease. AP = acute pancreatitis, CP = chronic pancreatitis, GERD = gastroesophageal reflux disease, MR = Mendelian randomization, SNP = single nucleotide polymorphisms.

Cochran’s *Q* test indicated no evidence of significant heterogeneity (*P* > .050). Both the MR-PRESSO global test (*P* > .050) and the MR-Egger intercept test (*P* > .050) suggested that there was no detectable horizontal pleiotropy in this MR study (Table 3). Finally, the funnel plot showed an approximately symmetrical distribution of SNPs used as genetic instruments for GERD, further supporting low heterogeneity (Fig. 8).

**Table 3:** The results of the sensitivity analysis in this MR analysis.

Outcome	Cochran’s *Q* test (IVW)		Egger Intercept test		MR-PRESSO
	*Q*	*P* value	Egger intercept	*P* value	*P* value of global test
AP (discovery cohort)	61.566	.754	0.003	.798	.779
AP (replication cohort)	65.483	.631	−0.008	.632	.656
CP (discovery cohort)	53.437	.886	0.010	.522	.892
CP (replication cohort)	60.118	.840	−0.062	.019	.840

AP = acute pancreatitis, CP = chronic pancreatitis, IVW = inverse-variance weighted, MR-PRESSO = Mendelian Randomization pleiotropy residual sum and outlier.

## 4. Discussion

This MR study comprehensively evaluated the causal association between GERD and pancreatitis, including both AP and CP. Using large-scale GWAS summary data, we found that genetically predicted GERD significantly increased the risk of AP and CP, indicating a potential causal relationship. The meta-analysis combining the discovery and replication cohorts further strengthened the robustness of this finding. Even after adjusting for cholelithiasis using multivariable MR (MVMR), the causal association between GERD and pancreatitis remained significant, suggesting that GERD is an independent risk factor. Furthermore, mediation analysis revealed that cholelithiasis partly mediated the causal pathway from GERD to pancreatitis, providing new insights into the complex interactions among gastrointestinal disorders.

The positive association between GERD and pancreatitis may be explained by several biological mechanisms. GERD is characterized by chronic reflux of gastric acid and digestive enzymes into the esophagus,^[36]^ which leads to mucosal inflammation and increased intra-abdominal pressure. These changes can impair duodenal motility and the function of the sphincter of Oddi, resulting in bile reflux, pancreatic duct obstruction, and premature activation of pancreatic enzymes. In addition, GERD is associated with alterations in the gastrointestinal microenvironment, including changes in acid secretion, bile acid metabolism, and intestinal microbiota composition.^[37-39]^ These alterations may induce oxidative stress, cytokine release, and systemic inflammation, thereby creating a proinflammatory milieu that predisposes individuals to pancreatitis.

From a metabolic perspective, GERD and pancreatitis share several overlapping risk factors, such as obesity, a high-fat diet, dyslipidemia, and insulin resistance.^[40-43]^ These metabolic abnormalities not only promote gastric reflux but also increase susceptibility to pancreatic injury. Elevated levels of inflammatory cytokines, including IL-6 and TNF-α,^[44,45]^ have been observed in GERD patients, and these cytokines are known to exacerbate pancreatic inflammation by promoting leukocyte infiltration, tissue edema, and acinar cell necrosis. Moreover, chronic GERD is often accompanied by enhanced oxidative stress and impaired antioxidant defense,^[46]^ which may contribute to pancreatic cell damage and fibrotic remodeling. Acid reflux may also cause duodenogastric reflux and reverse flow of bile acids into the pancreatic duct, disturbing the local pH and triggering intracellular Ca^2+^ signaling that promotes abnormal trypsinogen activation – a critical event in the early stages of pancreatitis. Additionally, GERD may indirectly affect the gut–pancreas axis by altering the secretion of intestinal hormones such as CCK and GLP-1, thereby influencing pancreatic exocrine function. Chronic inflammation induced by GERD^[47]^ may further enhance systemic immune activation, promoting the transition from acute to chronic pancreatic injury. Collectively, GERD may contribute to pancreatitis through mechanical obstruction, metabolic dysregulation, bile acid imbalance, and immune activation, reflecting the multifactorial nature of this causal link.

Our MVMR analysis indicated that the relationship between GERD and pancreatitis persisted even after controlling for cholelithiasis, implying that GERD promotes pancreatitis through non-gallstone-related mechanisms. However, the mediation analysis demonstrated that a proportion of the total effect of GERD on pancreatitis was indirectly mediated through cholelithiasis, suggesting an intertwined pathophysiological pathway. It is plausible that GERD increases the risk of gallstone formation via shared metabolic and inflammatory pathways. Both conditions are linked to obesity, lipid metabolism abnormalities, and bile acid dysregulation. GERD-induced alterations in bile composition or motility may facilitate cholesterol crystallization and gallstone formation, which in turn obstructs the common bile duct or pancreatic duct, triggering pancreatitis. Therefore, this study provides novel genetic evidence supporting the partial mediating role of cholelithiasis in the GERD-pancreatitis causal pathway.

From a clinical perspective, our findings have significant implications. Pancreatitis is a serious digestive disorder with substantial morbidity and mortality, and preventive strategies are essential. The observed causal relationship suggests that patients with GERD may benefit from active monitoring of pancreatic and biliary health. Early management of GERD, weight control, and surveillance for gallstone formation could potentially reduce the risk of developing pancreatitis. Moreover, clinicians should recognize the metabolic and inflammatory overlap between these conditions and consider integrated therapeutic approaches targeting shared biological pathways, such as anti-inflammatory or bile acid-modulating therapies.

Despite several strengths, including a large sample size, strict selection of instrumental variables, and multiple sensitivity analyses confirming the robustness of our findings, certain limitations should be acknowledged.^[48]^ First, all GWAS datasets were derived from European populations, which may limit generalizability to other ethnic groups. Second, MR analyses rely on the assumption that genetic variants affect the outcome solely through the exposure. Although no substantial pleiotropy or heterogeneity was detected, residual pleiotropy cannot be entirely excluded. Finally, the mediation analysis was based on summary-level data, which may not fully capture complex biological interactions. Future studies integrating individual-level data and experimental validation are warranted to further elucidate the mechanistic role of cholelithiasis in the GERD-pancreatitis pathway.

In summary, this MR study provides robust genetic evidence supporting a causal effect of GERD on both acute and chronic pancreatitis. Cholelithiasis partly mediates this relationship, suggesting that GERD may contribute to pancreatitis through both direct and indirect pathways. These findings enhance our understanding of the shared mechanisms underlying upper gastrointestinal and pancreatic diseases and offer new insights for early prevention and clinical management.

## 5. Conclusion

This MR study provides genetic evidence supporting a causal association between GERD and an increased risk of both AP and CP. Furthermore, mediation analysis suggests that cholelithiasis may partially mediate this relationship, highlighting its potential role in the GERD-pancreatitis pathway. These findings enhance our understanding of the shared pathogenic mechanisms between upper gastrointestinal and pancreatic diseases and underscore the importance of managing GERD and cholelithiasis to reduce the risk of pancreatitis.

## Author contributions

**Conceptualization:** Jinqiang Zhang.

**Writing – original draft:** Jinfu Zeng, Zhenhua Huang, Jinqiang Zhang.


